# Preparative Separation of Phenolic Compounds from *Chimonanthus praecox* Flowers by High-Speed Counter-Current Chromatography Using a Stepwise Elution Mode

**DOI:** 10.3390/molecules21081016

**Published:** 2016-08-04

**Authors:** Huaizhi Li, Yongqing Zhang, Qian Liu, Changlei Sun, Jia Li, Peng Yang, Xiao Wang

**Affiliations:** 1 College of Pharmacy, Shandong University of Traditional Chinese Medicine, 4655 Daxue Street, Jinan 250355, Shandong, China; huaizhili123@sina.com (H.L.); zyq622003@126.com (Y.Z.); scllbz@163.com (C.S.); 2 Key Laboratory of TCM Quality Control Technology, Shandong Analysis and Test Center, Shandong Academy of Sciences, 19 Keyuan Street, Jinan 250014, Shandong, China; 18765878227@126.com; 3 Kangsen Sanfeng Biological Engineering Technology Co., Jinan 250014, Shandong, China; Pengy@sina.com

**Keywords:** preparative separation, phenolic compounds, *Chimonanthus praecox* flower, high-speed counter-current chromatography (HSCCC), stepwise elution mode

## Abstract

High-speed counter-current chromatography (HSCCC) has been successfully used for the separation of eight compounds from *Chimonanthus praecox* flowers. Firstly, the crude extract of *Chimonanthus praecox* flowers was dissolved in a two-phase solvent system composed of petroleum ether–ethyl acetate–methanol–H_2_O (5:5:3:7, *v/v*) and divided into two parts: the upper phase (part I) and the lower phase (part II). Then, HSCCC was applied to separate the phenolic acids from part I and part II, respectively. Considering the broad polarity range of target compounds in part I, a stepwise elution mode was established. Two optimal solvent systems of petroleum ether–ethyl acetate–methanol–H_2_O–formic acid (FA) (5:5:3:7:0.02, 5:5:4.3:5.7:0.02, *v*/*v*) were employed in this separation. Five phenylpropanoids and two flavonoids were successfully separated from 280 mg of part I, including 8.7 mg of 3,4-dihydroxy benzoic acid (**a**, 95.3% purity), 10.9 mg of protocatechualdehyde (**b**, 96.8% purity), 11.3 mg of *p*-coumaric acid (**c**, 98.9% purity), 12.2 mg of *p*-hydroxybenzaldehyde (**d**, 95.9% purity), 24.7 mg of quercetin (**e**, 97.3% purity), 33.8 mg of kaempferol (**f**, 96.8% purity), and 24.6 mg of 4-hydroxylcinnamic aldehyde (**g**, 98.0% purity). From 300 mg of part II, 65.7 mg of rutin (**h**, 98.2% purity), 7.5 mg of 3,4-dihydroxy benzoic acid (**a**, 77.4% purity), and 4.7 mg of protocatechualdehyde (**b**, 81.6% purity) were obtained using the solvent system EtOAc–*n*-butanol (*n*-BuOH)–FA–H_2_O (4:1:0.5:5, *v*/*v*). The structures of the eight pure compounds were confirmed by electrospray ionization-mass spectrometry (ESI-MS), ^1^H-NMR and ^13^C-NMR. To the best of our knowledge, compounds **a**–**d** and **f** were the first separated and reported from the *Chimonanthus praecox* flower extract.

## 1. Introduction

*Chimonanthus praecox* (L.) Link (Wintersweet) is a popular potted, garden, cut-flower plant and landscape-design material in most countries for its strong fragrance, long blooming period, and unique flowering time [1,2,3,4]. Its flowers are important in traditional medicine in China, and have been used for the treatment of chest tightness, heatstroke, scald, bruise, and particularly as a cough expectorant [5,6,7]. Recent research shows that the phenolic compounds of *Chimonanthus praecox* flower (CPF) extract improve the body’s immune function and efficiently remove O_2_^−^, •OH, 1, 1-diphenyl-2-picrylhydrazyl (DPPH•) [8,9]. This implies that CPF may play a role in anti-aging, anti-inflammatory, and antioxidation. The main compounds of CPF are volatile oil and phenolic compounds. However, most pharmaceutical research of CPF focused on the volatile oil and few on phenolic compounds [10,11,12,13,14]. Modern pharmacological research showed that these phenolic compounds have extensive activities, such as antioxidant, anti-inflammatory, cardiovascular protection, and so on [15,16,17]. For these reasons, establishing an efficient and rapid method to separate and identify the phenolic compounds is needed for studies of the pharmacological and clinical effects of CPF.

**Figure 1:**
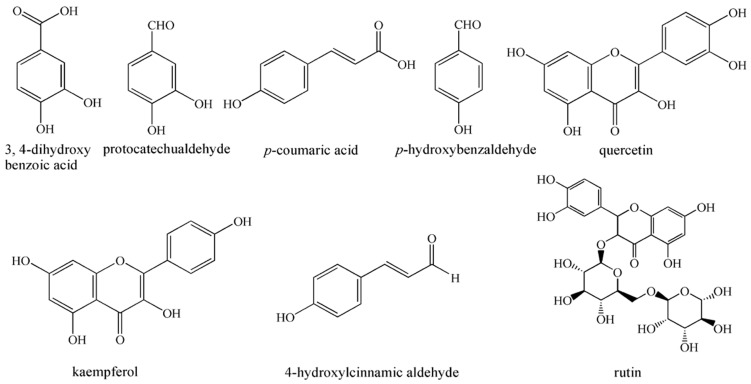
The chemical structures of eight phenolic compounds from *Chimonanthus praecox* flowers.

According to the reports, silica gel column chromatography and the preparative HPLC had been used for the separation and purification of several phenolic compounds from CPF [18]. However, these methods were tedious and they usually offered low recoveries. High-speed counter-current chromatography (HSCCC) without a solid support matrix is a preparative liquid–liquid chromatography, which could eliminate the irreversible adsorptive loss of samples. It enables the separation of the target compounds according to their partition coefficient. With high sample recovery rate, high speed, high loading, and relative simplicity [19,20], HSCCC has been widely used for the separation and purification of active components from natural products [21,22,23,24,25,26]. Additionally, it is a big challenge to separate the target components from the crude extract by HSCCC in a single run, because of the broad polarity range and complex components. Therefore, stepwise elution mode was employed in HSCCC to address this problem. Finally, eight highly pure compounds were successfully separated and purified from the CPF extract (Figure 1). As far as we know, the separation of phenolic compounds from CPF by HSCCC is reported here for the first time.

## 2. Results and Discussion

### 2.1. Selection of Two-Phase Solvent System

In HSCCC separation, the selection of a suitable solvent system is the first and most important step. The partition coefficient (*K_D_*) is expressed as the mass concentration of the target compound in the stationary phase divided by that in the mobile phase. In order to obtain a rapid and efficient separation, the optimal range of *K_D_* values is 0.5 to 2.0 [19,20].

**Figure 2:**
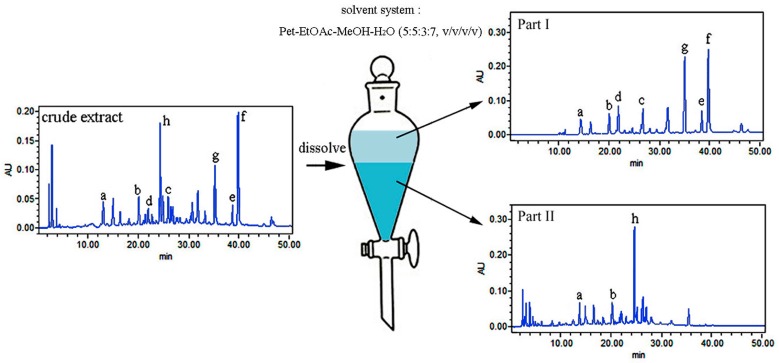
HPLC chromatograms of part I, part II, and the Chimonanthus praecox flower (CPF) extract. Pet: petroleum ether; EtOAc: ethyl acetate; MeOH: methanol. **a**. 3,4-dihydroxy benzoic acid, **b**. protocatechualdehyde, **c**. *p*-coumaric acid, **d**. *p*-hydroxybenzaldehyde, **e**. quercetin, **f**. kaempferol, **g**. 4-hydroxylcinnamic aldehyde, **h**. rutin.

According to the CPF extract profile of UV max absorption characteristics, phenolic compounds were identified to be the main component. Considering the polarity of phenolic compounds and the experience of separating phenolic compounds using HSCCC, several solvent systems composed of petroleum ether (Pet)–ethyl acetate (EtOAc)–methanol (MeOH)–H_2_O with different volume ratios (5:5:6:4, 5:5:5:5, 5:5:3:7, *v*/*v*) were investigated. As shown in Table 1, none of these solvent systems were suitable to separate the target compounds in one step. The crude extract contains complex components, and their polarities are wide, which brings certain difficulties for their separation. Fortunately, from Table 1, we can see that compounds **a**–**g** mainly distributed in the upper phase, while most of the impurities and target compound **h** were allocated to the lower phase in the solvent system Pet–EtOAc–MeOH–H_2_O (5:5:3:7, *v*/*v*). By this token, the solvent system could be used to pretreat the crude extract to achieve the preliminary purification. Therefore, the crude extracts were dealt with the solvent system Pet–EtOAc–MeOH–H_2_O (5:5:3:7, *v*/*v*) and divided into two parts: the upper phase (part I) and the lower phase (part II), which were used for further separation by HSCCC. The HPLC chromatograms are shown in Figure 2.

**Figure 3:**
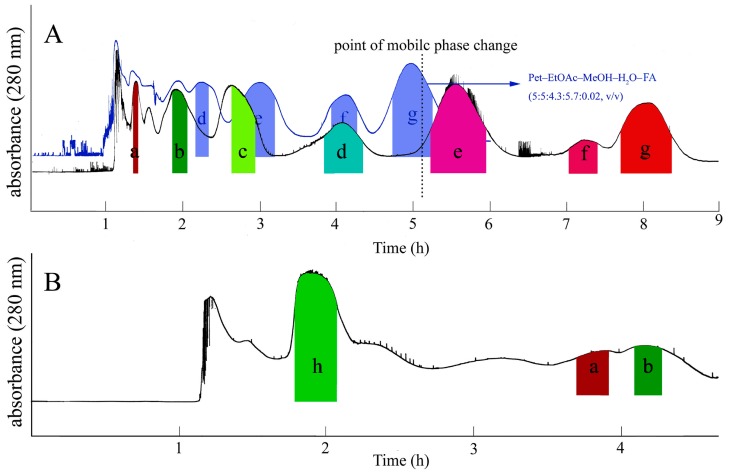
High-speed counter-current chromatography (HSCCC) Chromatograms of part I and part II. (**A**) Experimental conditions of part I: the black line HSCCC Chromatogram: solvent system: Pet–EtOAc–MeOH–H_2_O–FA (5:5:3:7:0.02, 5:5:4.3:5.7:0.02, *v*/*v*) in gradient elution. The stationary phase was the upper phase of Pet–EtOAc–MeOH–H_2_O–FA (5:5:3:7:0.02, *v*/*v*); the mobile phase was the lower phase of Pet–EtOAc–MeOH–H_2_O–FA (5:5:3:7:0.02, *v*/*v*) in 0–5 h, and the lower phase of Pet–EtOAc–MeOH–H_2_O–FA (5:5:4.3:5.7:0.02, *v*/*v*) in 5–9 h; UV detection wavelength: 280 nm; sample size: 280 mg; flow rate: 2 mL/min; revolution speed: 850 rpm. The solvent system of blue line HSCCC Chromatogram: Pet–EtOAc–MeOH–H_2_O–FA (5:5:4.3:5.7:0.02, *v*/*v*); (**B**) Experimental conditions of part II: Solvent system: EtOAc–*n*-BuOH–FA–H_2_O (4:1:0.5:5, *v*/*v*); Mobile phase: Lower phase; UV detection wavelength: 280 nm; Sample size: 300 mg; flow rate: 2 mL/min; Revolution speed: 850 rpm. **a**. 3,4-dihydroxy benzoic acid, **b**. protocatechualdehyde, **c**. *p*-coumaric acid, **d**. *p*-hydroxybenzaldehyde, **e**. quercetin, **f**. kaempferol, **g**. 4-hydroxylcinnamic aldehyde, **h**. rutin.

For separation of part I, a series of solvent systems were tested, but none of the solvent systems could provide suitable *K_D_* values (compounds **a**–**g**) between 0.5 and 2 at the same time, as shown in Table 1. Because of the wide polarity range of the target compounds, part I could not be separated using a single solvent system. So, stepwise elution mode will be a good choice in the HSCCC separation. The *K_D_* values of compounds **a**–**g** are shown in Table 1. The solvent system Pet–EtOAc–MeOH–H_2_O–FA (formic acid) (5:5:3:7:0.02, *v*/*v*) could provide suitable *K_D_* values for the separation of target compounds **a**–**e**. However, it was unsuitable for target compounds **f** and **g**. Therefore, the ratio of MeOH should be increased to reduce the *K_D_* values of the target compounds **f** and **g**. Although the solvent system Pet–EtOAc–MeOH–H_2_O–FA (5:5:4.3:5.7:0.02, *v*/*v*) could provide suitable *K_D_* values for compounds **e**–**g**, compounds **a**–**d** were eluted quickly, which resulted in poor resolution. As shown in Figure 3A, the blue line HSCCC Chromatogram.

According to these results, stepwise elution mode was used by combining the selected solvent systems in one single run, which involved two steps: the stationary phase was the upper phase of Pet–EtOAc–MeOH–H_2_O–FA (5:5:3:7:0.02, *v*/*v*), the mobile phase was the lower phase of Pet–EtOAc–MeOH–H_2_O–FA (5:5:3:7:0.02, *v*/*v*) until compound **a**–**d** was eluted out. Then, the mobile phase was switched to the lower aqueous phase of the solvent system Pet–EtOAc–MeOH–H_2_O–FA (5:5:4.3:5.7:0.02, *v*/*v*) to elute the other three compounds. Finally, seven pure compounds were obtained in a single run using the stepwise elution mode as shown in Figure 3A.

For the separation of part II, the solvent system composed of EtOAc–EtOH–FA–H_2_O (4:1:0.1:5, *v*/*v*) were first tested, but all of the compounds were eluted too quickly. So, the solvent system was adjusted by replacing EtOH with *n*-butanol (*n*-BuOH) to achieve efficient separation. The *K_D_* values of compound **h** are shown in Table 1. When the solvent system EtOAc–*n*-BuOH–FA–H_2_O (4:1:0.5:5, *v*/*v*) was employed, compounds **h**, **a**, and **b** were separated successfully. The HSCCC chromatogram of part II is shown in Figure 3B.

### 2.2. HSCCC Separation

As shown in Figure 3A, part I was successfully separated with the Pet–EtOAc–MeOH–H_2_O–FA (5:5:3:7:0.02, 5:5:4.3:5.7:0.02, *v*/*v*) solvent system by stepwise elution mode. The separation time was within 9 h and the retention of the stationary phase was 58%. Seven kinds of compounds were obtained from 280 mg of part I, including 8.7 mg of 3,4-dihydroxy benzoic acid (**a**), 10.9 mg of protocatechualdehyde (**b**), 11.3 mg of *p*-coumaric acid (**c**), 12.2 mg of p-hydroxybenzaldehyde (**d**), 24.7 mg of quercetin (**e**), 33.8 mg of kaempferol (**f**), and 24.6 mg of 4-hydroxylcinnamic aldehyde (**g**). Figure 3B shows that the EtOAc–*n*-BuOH–FA–H_2_O (4:1:0.5:5, *v*/*v*) solvent system was employed to separate part II. The completion time of separation was within 4.5 h, and the stationary phase retention was about 51%. From 300 mg of part II, 65.7 mg of rutin (**h**), 7.5 mg of 3,4-dihydroxy benzoic acid (**a**), and 4.7 mg of protocatechualdehyde (**b**) were obtained.

### 2.3. HPLC Analysis of Crude Extract and HSCCC Peaks

Figure 2 shows the HPLC chromatogram of part I, which mainly presented seven phenolic compounds (peaks a–g) corresponding to 3,4-dihydroxy benzoic acid (**a**), protocatechualdehyde (**b**), *p*-coumaric acid (**c**), *p*-hydroxybenzaldehyde (**d**), quercetin (**e**), kaempferol (**f**), and 4-hydroxylcinnamic aldehyde (**g**), in sequence based on the peak area normalization method at the optimized detective wavelength of 280 nm. Peak a represented 5.5% of the total peak area and peaks b–g accounted for 6.3%, 7.0%, 7.2%, 6.4%, 23.9%, and 22.7%, respectively. For part II, where three phenolic compounds (peaks a, b, and g) corresponded to 3,4-dihydroxy benzoic acid (**a**), protocatechualdehyde (**b**), and rutin (**h**). Peak a, b, and g represented 4.7%, 5.8%, and 18.1%, respectively.

**Figure 4:**
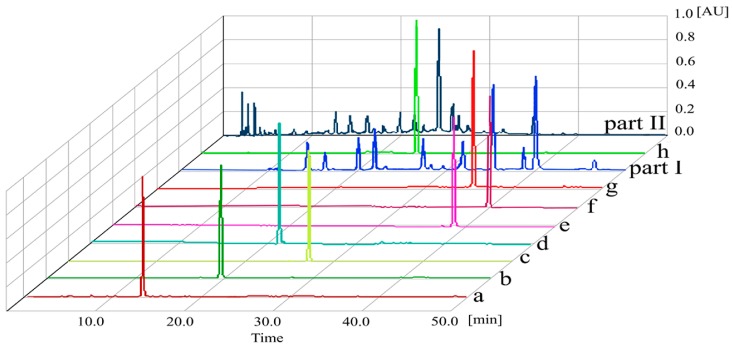
HPLC chromatograms of HSCCC peak fractions (a–h), part I and part II. Conditions: column, Waters Symmetry Shield™ RP18 column (250 mm × 4.6 mm i.d., 5 μm); mobile phase, acetonitrile (**A**) 0.2% formic acid aqueous solution (**B**) (0–50 min, 5%–60% **A**); Column temperature, 25 °C; Detection wavelength, 280 nm; Injection volume, 20 µL; Flow rate, 1 mL/min. **a**. 3,4-dihydroxy benzoic acid, **b**. protocatechualdehyde, **c**. *p*-coumaric acid, **d**. *p*-hydroxybenzaldehyde, **e**. quercetin, **f**. kaempferol, **g**. 4-hydroxylcinnamic aldehyde, **h**. rutin.

Figure 4 shows the HPLC chromatograms of peaks separated by HSCCC, which demonstrates that the phenolic compounds of part I were all obtained with purities of over 95.3%. For part II, the isolated peaks (peaks a, b, and g) were obtained with the purities of 98.2%, 77.4%, and 81.6%, respectively.

## 3. Experiment Section

### 3.1. Apparatus

The HSCCC instrument in the present study was a TBE-300A (Tauto Biotech, Shanghai, China), equipped with three multilayer coil columns (tube diameter: 1.6 mm) with total volume of 300 mL. The rotation speed could be controlled from 0 to 1000 rpm. The two phases were pumped by a constant flow pump (Beijing Heng Odd Instrument Co., Ltd., Beijing, China), and the effluent was continuously monitored with a XRS-HD21-1 detector (Beijing Sirius Instrument Co., Ltd., Beijing, China) operating at 280 nm. A DLSB-5 constant temperature circulator (Zhengzhou Yucheng Instrument Co., Ltd., Zhengzhou, China) was used to control the temperature at about 25 °C. A model STR1001 portable recorder (Fuji Electric Co., Ltd., Shanghai, China) was employed to record the chromatogram.

The crude extract and samples were analyzed using the Waters Alliance 2695 series (Waters, Milford, MA, USA) made in USA, equipped with a four binary gradient pump, Photodiode Array Detector (PDA), and autosampler.

### 3.2. Reagents and Materials

HSCCC solvents, including ethyl acetate (EtOAc), petroleum ether (Pet), *n*-butanol (*n*-BuOH), ethanol (EtOH), and methanol (MeOH) were analytical grade, purchased from Fuyu Chemical Factory (Tianjin, China). Formic acid (FA, Kermel Chemical Reagent Co. Ltd., Tianjin, China) and acetonitrile (Fisher Scientific Company, Fair Lawn, NJ, USA) used for HPLC analysis were of chromatographic grade. All water used in this study was produced by an Elga Purelab water system (Elga, High Wycombe, UK).

Fresh flowers of *Chimonanthus praecox* were collected from the courtyard of Shandong Academy of Sciences (Jinan, China) in February 2015, and identified by Professor Jia Li (Shandong University of Traditional Chinese Medicine, Jinan, China). A voucher specimen (No. 20150222) was deposited in Shandong Analysis and Test Center, Shandong Academy of Sciences.

### 3.3. Preparation of Sample

Fresh flowers of *Chimonanthus praecox* (0.8 kg) were refluxed with 2 L of 70% ethanol three times (each for 2 h). The extract solutions were filtered before being combined, and were concentrated under reduced pressure at 50 °C until the ethanol was removed. Then, it was diluted with 2 L water and extracted by Pet and EtOAc three times. Thus, 6.7 g of EtOAc extract were obtained. The extraction yield of EtOAc extract was 0.84%. The EtOAc extract was dissolved in a separator funnel with 700 mL of the solvent system composed of Pet–EtOAc–MeOH–H_2_O (5:5:3:7, *v*/*v*). The upper and lower phases were divided into two parts. The upper phase was collected and evaporated to dryness under vacuum, and 3.37 g of powder (part I) was obtained. The lower phase was also collected and evaporated to dryness under vacuum, and 3.15 g of powder (part II) was obtained. Both parts were stored in the refrigerator (4 °C) and further separated by HSCCC. The HPLC chromatograms of the EtOAc extract, part I, and part II are shown in Figure 2.

### 3.4. Determination of Partition Coefficient (K_D_ Value)

In order to achieve effective separation of the target compounds from the CPF extract, the *K_D_* values of various solvent systems composed of EtOAc–*n*-BuOH–FA–H_2_O and Pet–EtOAc–MeOH–H_2_O–FA with different volume ratios were measured by HPLC as follows. Two milliliters of each phase of the solvent systems were prepared in a test tube. About 3 mg of sample was dropped into a tube and fully shaken to thoroughly mix. After equilibrium, the upper and the lower phases were separated completely, and each phase was injected to HPLC. The *K_D_* values of target components are defined as *K_D_* = *A_U_/A_L_*. *A_U_*: The HPLC peak area of the target components in the upper phase; *A_L_*: The HPLC peak area of the target components in the lower phase.

### 3.5. Separation Procedure

The HSCCC was operated as follows. Firstly, the whole column was entirely filled with the upper stationary phase at 20 mL/min. When the rotation speed was controlled at 850 rpm, the lower phase was pumped into the column at 2 mL/min. After the steady hydrodynamic equilibrium was established, the sample solution was injected into the separation system through the injection port. The eluent was continuously detected by UV monitor at 280 nm. The peak fractions were manually collected according to the UV absorbance profile and analyzed by HPLC. When the separation was finished, stationary phase retention was measured by forcing the column contents into a measuring vessel with pressurized air.

### 3.6. HPLC Analysis

The purified compounds from the HSCCC separation and the EtOAc extract, as well as parts I and II, were analyzed by HPLC with a Waters Symmetry Shield™ RP18 column (250 mm × 4.6 mm, i.d., 5 μm) at 25 °C. The mobile phase was acetonitrile (solvent A) and 0.2% formic acid aqueous solution (solvent B) in a gradient mode (5%–60% A, 0–50 min). The flow-rate was 1.0 mL/min and the detection wavelength was 280 nm.

### 3.7. Identification of the Isolated Compounds

After the purified compounds was evaporated to dryness under vacuum, each compound was dissolved in DMSO for analysis by nuclear magnetic resonance (NMR) with a Varian-400 spectrometer and electrospray ionization-mass spectrometry (ESI-MS) on an Agilent 1100/MS-G1946 (Agilent, Santa Clara, CA, USA). The detailed data of each compound are as follows:

Compound (**a**) (peak a in Figure 3A): Positive ESI-MS, *m*/*z* 155 [M + H]^+^. ^1^H-NMR (DMSO-*d*_6_, 400 MHz) δ: 7.33 (1H, d, *J* = 2.0 Hz, H-2), 7.29 (1H, dd, *J* = 2.0, 8.0 Hz, H-6), 7.28 (1H, d, *J* = 8.0 Hz, H-5). ^13^C-NMR (DMSO-*d*_6_, 100 MHz) δ: 115.5 (C-5), 117.0 (C-2), 122.1(C-1), 122.3 (C-6), 145.3 (C-3), 150.3 (C-4), 167.6 (-COOH). Compared with the reported date [27], compound **a** was identified as 3,4-dihydroxy benzoic acid.

Compound (**b**) (peak b in Figure 3A): Positive ESI-MS, *m*/*z* 139 [M + H]^+^. ^1^H-NMR (CD_3_OD, 400 MHz) δ: 9.67 (1H, brs, -CHO), 7. 29 (1H, dd, *J* = 1.6, 7.2 Hz, H-6), 7.27 (1H, d, *J* = 1.6 Hz, H-2), 6.92 (1H, d, *J* = 7.8 Hz, H-5). ^13^C-NMR (CD_3_OD, 100 MHz) δ: 115.6 (C-5), 116.5 (C-2), 126.4 (C-6), 129.9 (C-1), 147.3 (C-3), 153.9 (C-4), 192.2 (-CHO). Compared with the reported date [28], compound **b** was identified as protocatechualdehyde.

Compound (**c**) (peak c in Figure 3A): Positive ESI-MS, *m*/*z* 165 [M + H]^+^. ^1^H-NMR (DMSO-*d*_6_, 400 MHz) δ: 7.51 (1H, d, *J* = 16.0 Hz, H-7), 7.50 (2H, d, *J* = 8.0 Hz, H-2, 6), 6.80 (2H, d, *J* = 8.5 Hz, H-3, 5), 6.30 (1H, d, *J* = 16.0 Hz, H-8). ^13^C-NMR (DMSO-*d*_6_, 100 MHz) δ: 115.8 (C-8), 116.2 (C-3, 5), 125.7 (C-1), 130.5 (C-2, 6), 144.6 (C-7), 160.1 (C-4), 168.5 (C-9). Compared with the reported date [29], compound **c** was identified as *p*-coumaric acid.

Compound (**d**) (peak d in Figure 3A): Positive ESI-MS, *m*/*z* 121 [M − H]^−^. ^1^H-NMR (DMSO-*d*_6_, 400 MHz) δ: 9.77 (1H, s, -CHO), 7.76 (2H, d, *J* = 8.4 Hz, H-2, 6), 6.93 (2H, d, *J* = 8.4 Hz, H-3, 5). ^13^C-NMR (DMSO-*d*_6_, 100 MHz) *δ:* 116.4 (C-3, 5), 128.6 (C-1), 132.6 (C-2, 6), 164.1 (C-4), 191.3 (-CHO). Compared with the reported date [30], compound **d** was identified as *p*-hydroxybenzaldehyde.

Compound (**e**) (peak e in Figure 3A): Positive ESI-MS, *m*/*z* 303 [M + H]^+^. ^1^H-NMR (DMSO-*d*_6_, 400 MHz) δ: 7.68 (1H, d, *J* = 1.7 Hz, H-2′), 6.90 (1H, d, *J* = 8.4 Hz, H-5′), 7.55 (1H, d, *J* = 1.7, 8.4 Hz, H-6′), 6.19 (1H, d, *J* = 1.6 Hz, H-6), 6.41 (1H, d, *J* = 1.6 Hz, H-8). ^13^C-NMR (DMSO-*d*_6_, 100 MHz) δ: 176.3 (CO), 164.4 (C-7), 161.2 (C-9),156.6 (C-5), 147.3 (C-2), 148.2 (C-4′), 145.5 (C-3′), 136.2 (C-3), 122.4 (C-1′), 120.4 (C-6′), 116.1 (C-5′), 115.5 (C-2′), 103.5 (C-10), 98.7 (C-6), 93.8 (C-8). Compared with the reported date [31], compound **e** was identified as quercetin.

Compound (**f**) (peak f in Figure 3A): Positive ESI-MS, *m*/*z* 287 [M + H]^+^. ^1^H-NMR (DMSO-*d*_6_, 400 MHz) δ: 6.38 (1H, d, *J* = 2.0 Hz, H-8), 6.19 (1H, d, *J* = 2.0 Hz, H-6), 6.89 (1H, d, *J* = 9.0 Hz, H-3′, 5′), 8.05 (2H, d, *J* = 9.0 Hz, H-2′, 6′). ^13^C-NMR (DMSO-*d*_6_, 100 MHz) δ: 94.1 (C-8), 98.6 (C-6), 103.9 (C-10), 115.8 (C-3′, 5′), 123.3 (C-1′), 130.1 (C-6′, 2′), 136.5 (C-3), 147.7 (C-2), 157.6 (C-9), 160.0 (C-4′), 162.0 (C-5), 165.1 (C-7), 176.7 (C-4). Compared with the reported date [30], compound **f** was identified as kaempferol.

Compound (**g**) (peak g in Figure 3A): Positive ESI-MS, *m*/*z* 148 [M + H]^+^. ^1^H-NMR (DMSO-*d*_6_, 400 MHz) δ: 9.74 (1H, d, *J* = 7.8 Hz, -CHO), 7.67 (2H, d, *J* = 8.3 Hz, H-2, 6), 6.55 (1H, dd, *J* = 7.8, 15.8 Hz, H-2′), 7.53 (1H, d, *J* = 15.8 Hz, H-1′), 7.41 (2H, d, *J* = 8.3 Hz, H-3, 5). ^13^C-NMR (DMSO-*d*_6_, 100 MHz) δ: 116.3 (C-3, 5), 128.8 (C-1), 129.7 (C-2′), 132.6 (C-2, 6), 157.2 (C-1′), 163.8 (C-4), 191.4 (-CHO). Compared with the reported date [32], compound **g** was identified as 4-hydroxylcinnamic aldehyde.

Compound (**h**) (peak h in Figure 3B): Positive ESI-MS, *m*/*z* 611 [M + H]^+^. ^1^H-NMR (DMSO-*d*_6_, 400 MHz) δ: 7.60 (1H, d, *J* = 2.0 Hz, H-2′), 6.35 (1H, d, *J* = 2.0 Hz, H-8), 7.56 (1H, dd, *J* = 2.0, 8.4 Hz, H-6′), 6.83 (1H, d, *J* = 8.4 Hz, H-5′), 6.18 (1H, d, *J* = 2.0 Hz, H-6), 5.06 (1H, d, *J* = 8.0 Hz, Glc-H-1′′), 4.46 (1H, brs, Rha-H-1′′), 1.04 (3H, d, *J* = 6.2 Hz, Rha-CH3). ^13^C-NMR (DMSO-*d*_6_, 100 MHz) *δ*: 18.3 (Rha-C-6′′′), 69.1 (Glc-C-6″), 69.5 (Rha-C-5′′′), 72.6 (Rha-C-3′′′), 72.8 (Rha-C-2′′′), 72.8 (Glc-C-4″), 74.2 (Rha-C-4′′′), 75.9 (Glc-C-2″), 78.7 (Glc-C-3″), 77.4 (Glc-C-5″), 94.3 (C-8), 99.8 (C-6), 104.9 (Glc-C-1″), 102.6 (Rha-C8m-1′′′), 104.9 (C-10), 117.1 (C-5′), 116.4 (C-2′), 123.6 (C-6′), 135.8 (C-3), 123.9 (C-1′), 146.1 (C-3′), 158.7 (C-9), 149.8 (C-4′), 159.2 (C-2), 166.3 (C-7), 179.8 (C-4), 163.4 (C-5). Compared with the reported date [30], compound **h** was identified as rutin.

## 4. Conclusions

In this report, an effective strategy was successfully established to separate eight compounds with purity over 95% from the CPF extract. The results of our study could demonstrate the following two points: (1) a preliminary purification using a suitable solvent system is necessary for the successful separation by HSCCC, especially with a complicated crude sample; (2) the stepwise elution for the separation of different polarities of target compounds by HSCCC is an efficient strategy. The present research could provide a rapid and efficient pattern for the separation and purification of phenolic acids from the EtOAc extract of *Chimonanthus praecox* flowers, and these methods could be applied to separate and purify other complicated compounds from natural products.

## Figures and Tables

**Table 1:** Partition coefficient (*K_D_*) of the target compounds in several solvent systems.

Sample	Solvent System (*v*/*v*)	Partition Coefficient *(K_D_)* ^b^							
		a	b	c	d	e	f	g	h
Crude extract ^a^	Pet–EtOAc–MeOH–H_2_O 5:5:6:4	0.11	0.37	2.53	1.97	1.03	8.62	>10	<0.1
	Pet–EtOAc–MeOH–H_2_O 5:5:5:5	0.21	0.58	3.01	2.79	1.38	>10	>10	<0.1
	Pet–EtOAc–MeOH–H_2_O 5:5:3:7	0.79	1.32	5.36	5.21	3.01	>10	>10	<0.1
Part I	Pet–EtOAc–MeOH–H_2_O–FA 5:5:4:6:0.02	0.17	0.25	0.43	0.99	1.26	1.77	2.37	–
	Pet–EtOAc–MeOH–H_2_O–FA 5:5:3:7:0.02	0.47	0.59	0.98	1.42	1.76	2.56	3.62	–
	Pet–EtOAc–MeOH–H_2_O–FA 5:5:4.2:5.8:0.02	0.23	0.37	0.28	0.94	0.97	1.35	1.83	–
	Pet–EtOAc–MeOH–H_2_O–FA 5:5:4.3:5.7:0.02	0.16	0.18	0.25	0.64	0.92	1.12	1.51	–
Part II	EtOAc–EtOH–FA–H_2_O 4:1:0.1:5	–	–	–	–	–	–	–	0.26
	EtOAc–*n*-BuOH–FA–H_2_O 4:1:0.1:5	–	–	–	–	–	–	–	0.47
	EtOAc–*n*-BuOH–FA–H_2_O 4:1:0.5:5	–	–	–	–	–	–	–	0.92

^a^ The crude extract refers to the fraction obtained after EtOAc extraction. ^b^ *K_D_ = A_U_/A_L_. A_U_*: The HPLC peak area of the target components in the upper phase; *A_L_*: The HPLC peak area of the target components in the lower phase. FA: Formic acid; *n*-BuOH: *n*-Butanol.
